# Bioethics as a language game: probing the quality of moral guidance in principlism

**DOI:** 10.1007/s11017-025-09702-9

**Published:** 2025-02-26

**Authors:** Matthew Vest

**Affiliations:** Department of Biomedical Education and Anatomy, Center for Bioethics and Medical Humanities, College of Medicine, The Ohio State University Wexner Medical Center, Columbus, Ohio USA

**Keywords:** Principlism, Language, Democratic reason, *Ascesis*, Beauchamp, Childress, Kant, Rawls, Wittgenstein

## Abstract

This essay asks what quality of moral guidance is offered via the language of principlism, the *lingua franca* of bioethics. In particular, I suggest three approaches to principlist language via Kant, Rawls, and Wittgenstein. A ‘top down’ Kantian view of language would seem to offer ‘pure’ or ‘crystalline’ moral guidance as autonomy, beneficence, non–maleficence, and justice function as linguistic links to draw us towards universal values *up* or *out there* to engage. While drawing upon Rawls, Beauchamp and Childress differ importantly by citing a universal morality grounded in reflective equilibrium amongst citizens. Principlism, hence, possesses a democratic form where the common morality depends upon a historically consistent majority position; what is ‘universal’ arises from political ‘bottom up’ discourses and processes. Wittgenstein, however, offers a notably different view of language that embraces the mystical and aesthetic realities of ‘the ethical’ while also affirming the grounding of language in everyday contexts. Not unlike the Stoics, language for Wittgenstein is ascetic in that it is a practice, a formative exercise that reveals the humility of language as an immanent ‘game’ that should nevertheless inspire one towards ‘the ethical.’

## Perspectives and expectations: unveiling antecedent epistemological commitments

Symptomatology (also called semiology) refers to the study of the indications, markers, and expressions of a disease or disorder. Symptomatology exemplifies a tension that arises within evidence-based medicine when attention is turned to the *dynamics* between a root cause and its expression, both of which are distinct and yet interrelated. On the one hand, symptoms are sufficient to identify most diseases, and on the other, the groups of physical feelings or ‘facts’ that appear related to a disease are not always easily readable. Symptoms vary person to person, and this complexity leads to symptomatology as the study and classification of symptoms.

The relationships between disease and symptom, pathology and symptomology, illustrate complexity within medicine, largely because they reveal something important about perspectives and expectations. Namely, what one seeks to discover impacts methodology and disciplines. As Polanyi noted [2], personal experiences and shared knowledge strongly influence scientific discoveries, and this pushes against notions of pure autonomous objectivity or crystalline neutrality in the sciences. Regardless of the latest cutting-edge of biotech, medicine remains a skilled method or way (*ars, artis)*, and a good physician will not eschew the nuances and dynamics between root problems and felt expressions as evidenced in symptomatology.

The point here in considering the dynamics between pathology and symptomology is simple: the perspectives, expectations, and viewpoints one brings to medicine and philosophy carry immense import. Each individual carries a particular narrative, and some more powerful narratives become collective frameworks that underscore research questions and the hypothesized answers they generate. At the heart of this, moreover, is language, at least in so far as the words one uses calls one to attend to the assumptions and backstories that inevitably ground frameworks of meaning. Wittgenstein attests to this succinctly—“to imagine a language is to imagine a form of life” [1, PI 19]—which I take to mean that the words one uses are an invitation to examine one’s lived habits as much as one’s language.

Enter Beauchamp and Childress’ *Principles of Biomedical Ethics,* now in its* 8th ed*. (and counting?). For many, this work represents the bible of bioethics. They may compare the four principles to the ten commandments as concise codifications of static moral axioms. In both, however, much work remains to uncover the depths of what is contained in so few words. One may call this work due diligence or authenticity for only an ersatz biblical scholar would suggest the ten commandments are a *self-explanatory* summary of the entire Levitical law. Similarly, to think that principlist language is self-explanatory fails on many levels, even while the *Principles of Biomedical Ethics* exhibits a “cool detached writing style that is expressed by an impersonal narrator” [3, p. 13]. The changes across the eight editions have been far from insignificant and impersonal as a cottage industry of publications responding to Beauchamp and Childress—*sicut vero*, this essay—engage, critique, and confront principlism for a disjunct that Marvin Lee aptly described as being “thick in status” yet “thin in content” [4, p. 525].

Danner Clouser and Bernard Gert’s “A critique of principlism” [5] was a particularly notable challenge to Beauchamp and Childress that probed whether the four principles functioned as substantive “guides to action” or rather were lesser “names” for a series of loosely associated matters. Andrew Lustig [6], David Degrazia [7], Henry Richardson [8], and many others have joined the scholarly dialogue, supporting or critiquing principlism; this is not to list the criticisms from casuist, feminist, religious, pragmatist, or other vantage points. Ironically, the more responses are leveled against Beauchamp and Childress, the more principlism’s impact seems to grow, leading to John Arras’ well-known comparison of *Principles of Biomedical Ethics* to Borg in *Star Trek: The Next Generation* [9]. A hive of cybernetically-enhanced humanoid drones, the Borg encounters opposition not by rejection but by assimilation: “No sooner do [critics of Beauchamp and Childress] launch a crippling broadside against the juggernaut of PBE…than their critique is promptly welcomed with open arms, trimmed of its perceived excesses, and incorporated into the ever-expanding synthesis of the next edition” [9, p. 12].

The aim of this essay differs from these critiques by focusing on language, and to follow the phenomenon of language surrounding principlism compels one to note firstly, *sine ira et studio,* the immense impact of Beauchamp and Childress. When language becomes so widely employed and common—whether via acceptance or rejection—a considered response is to pause and consider ‘why’ it has become so successful. This contemplative pause allows one to note layers of epistemological ambiguity inherent within one’s use of words. For example, as principlist debates rounded the quarter century mark, Richard Davis [10] flagged core differences in 1995 between Beauchamp and Childress’ and Clouser and Gert’s “expectations for a ‘theory’” [10, p. 103–104]. Do the principles derived from a moral theory need to be directive and systematically related to one another as Clouser and Gert’s foundationalism expects? Or, in line with principlism, are the demands of a theory less rigorous, allowing for a coherentist understanding? These are critical epistemological differences that virtually guarantee winning conclusions for both ‘sides’ based on their own respective definitions and expectations. But to what end? A debate won via rival “antecedent epistemological commitments” [10, p. 85] can only be as beneficial as conversing alone in an echo canyon.

Moreover, it is difficult to miss the ironic correlation between principlism’s epistemological ambiguity and its immense influence. In a realpolitik sense, ambiguity as well as generalized collectives have an advantage, allowing a political slogan, mantra, or association to align with large numbers of people despite substantive differences on the individual level. On the other hand, beyond realpolitik, aspirational visions arise, leading to individuals to seek clarity and a description (vs. *explanation*) of everything that is simply “put before us” [1, PI 126].^1^ Where Davis rightly directs one to consider epistemological differences, language offers a way to do just that, and it is here that I propose to trace three particular approaches to language that commonly frame principlist discussion in bioethics. The first is that language works in a ‘top down,’ scientific manner, and while I do not examine Kant’s philosophy of language or debates regarding his possible neglect of language [11], Kant is a fitting representative figurehead for scientific language. The second is that language works best within a ‘bottom up’ democratic rationality; Rawls is the natural figurehead for this view, who develops and modifies Kantian practical reason. The last approach to language comes from Wittgenstein’s ascetic framework that sees language as both a practice and exercise remaining close to the ground while simultaneously aiming upward. In short, the aim of this essay is not to join the direct philosophical challenges to principlism from a particular vantage point, but rather to draw attention to how expectations within language inevitably influence methods and ends in medicine and bioethics. This is, as the introduction to this special issue has put it, a way to attune to one’s ‘ways with words.’

## Scientific language: Kantian roots undergirding principlism

To clarify from the start, the primary connection between science and language I am proposing is not inherent or essential to science or language. The point is not to engage a metaphysics of science or language as much as to note attitudes and expectations for science, however *well* or *misplaced,* that carry over to language. Hence, far from a critique of natural science as a discipline, scient*ism* or scientistic thinking is my primary concern, just as a parallel concern with philosophy or language is not something inherent within these activities as much as one’s expectations and postures often surpass the possibilities of these activities. Paul Horwich summarizes this nicely, noting the tendency to be captivated by “the expectation that philosophy can and should deliver a priori theories that—like good scientific theories—are simple, unifying, and explanatory” [12, p. 15].

Noting the limits of scientific knowledge should be nothing new. The work of experienced scientists and physicians bring to light that research is on-going; empirical research, by definition, assumes incompletion. For all the cultural momentum and sociological phenomena behind the mantra ‘follow the science,’ the reality is that such enthusiasm and naiveite often demeans the real work of science. The empirical, data-driven, evidence-based pathways of science and medicine yield remarkable goods, and yet to ask for ‘cure more than care’ in medicine sets the bar unrealistically high. Equally, one might also note with nuclear physicist Alvin Weinberg the tendency to ask *political* questions of science as though science can answer concretely whether one should or should not act upon the abilities of science and technology [13]. Whereas geothermal physics describes possibilities for drilling oil in the Antarctic, deciding to act or not upon this possibility moves beyond the bounds of science. Weinberg criticizes such over simplicity: “many of the issues which arise in the course of the interaction between science and technology and society—e.g., the deleterious side effects of technology, or the attempts to deal with social problems through the procedures of science—hang on the answers to science which can be asked of science and yet *which cannot be answered by science*” [13, p. 210]. Hence Weinberg’s term, “trans–science,” summarizes questions asked of science that epistemologically transcend science.

Moreover, the impulse of trans–science shares a family resemblance with Kant’s a priori knowledge (*scientia*) as a narrowed, reductionist view of knowledge. The broader Enlightenment lineage of Descartes, Locke, and Hume warrants attention here, and yet Kant in particular champions the placement of metaphysics “upon the true path of science,” and the assumption behind this maneuver is that “theory” and “theorizing” is the best path to understand reality [14, p. 8]. This theorizing lies at the heart of Wittgenstein’s critique of modern philosophy, a philosophy that presumes the sense of world is best known through the methods of science and technology. Jean-Yves Lacoste describes this path of modern science as a reduction and fragmentation in contrast with ancient *theoria* and *ethos*; the depth of cosmological and theological meaning in the ancient view of *phusis* is lost amidst the cares of physical science, leaving only logic in place of *Logos*. He writes:

*Logos* alone remains, then, and it is no longer anything but the *logos* of logic and the surveillance that it exercises over scientific procedures. All philosophy faithful to its origins ought to protest against its reduction to the state of *Wissenschaft*: its proper rigor does not have to be that of the sciences, even if it ought not be any less [15, pp. 11–12].

It would be an overstatement to claim that the reduction of *logos* to logic or *phusis* to physical science *is* Kant’s project, and yet one should not miss how the Kantian a priori space paves the way for this reductionism [17, pp. 55–68]. Kant primarily aims to investigate the possibility both of synthetic a priori judgements [17, B 19] and metaphysics as a science [17, B 22] for the sake of transcendental proof. He asserts that the mode of “sensible representation (intuition)” necessarily belongs to one’s consciousness, and that this mode precedes knowledge of objects as the “intellectual form” of such knowledge. Hence, Kant’s formal a priori knowledge inaugurates necessary “thought (categories).” Accordingly, transcendental idealism—“pure” and “necessary” concepts—emerge within individuals as essential ways of ordering the world [17, A 129–130].

The key to connecting this discussion to principlism lies in Kants’ assumption that prior to, or independently of, experience, the natural world is conceivable. Hence, if and when this mentalist framework is assumed with the language of principlism, the four principles will be approached with a sort of sacred, transcendental value. Thus, a Kantian principlist may begin by *asserting* autonomy or beneficence or justice as objective realities, when really, they are only describing a priori cognitive structures. Hence as Fergus Kerr notes, this Kantian space presents metaphysics, as a science of being, in a magical way where invoking certain words or terms calls out some transcendental meaning [18, pp. 250–254]. It is true that Kant aimed at conceptual knowledge of ‘things’ in nature, but even so, the emphasis of synthetic a priori knowledge compels one towards a priority and expectation of mentalist theorizing.^2^

Is Kant’s scientific thinking essential to principlist language? Again, that would be misstating the case. The point, rather, is to draw out the ramifications when Kant’s scientific thinking is the framework many bring to principlism. Imagine a 1st or 2nd year medical student with little to no humanities background who transitions from intensive basic sciences to a single lecture on medical ethics. How is the student to think when the four principles appear on a slide presentation? Should one be surprised at all when medical students write short reflections that simply assert the term ‘autonomy’ as *the* basis for a clinical ethics position and suppose that the job is done? Or what of the comment a physician-bioethicist once made to Arras: “This [principlism method] is what our student-doctors need. It’s really objective, based on principles, *just like a science*” [3, p. 10, emphasis added]. Again, when this scientific and transcendental attitude emerges, it is well and good to point out “well, this isn’t *really* Kant at all,” but, the de facto reality remains that when bioethics students or even clinical ethicists expect and presuppose that bioethics provides generally applicable, universally accepted guidelines akin to Kant’s transcendental idealism, one is seeing evidence for Tom Koch’s thesis that bioethics has indeed stolen medicine, uprooting medicine from “the broader framework of social conscience and development” [19, p. xix].

This scientific image of language presumes a ‘top-down’ framework. Some repository of meaning transcendentally ‘out there’ magically guides one towards a theoretical meaning, removed from traction with the actual landscape under one’s feet. This view of language aligns with a type of metaphysical self–understanding that emerges throughout modern philosophy: *rationality-as-representation* [16, p. 71–101]. Such theorizing rationality assumes that one’s mental conceptions are real representations of reality; if only one mentally conceives rightly, one mentally conceives reality. Then, *theorize* about autonomy and justice rightly, and one is almost there. Hence ambiguous theorizing statements take the form of a scientific claim: *this is how things are*. In the next section, I will draw out a modification on Kant via Rawl’s democratic rationality and language. It is fitting to transition by first noting with Richard Read the pattern of Kantian scientistic thinking manifest in the opening sentence of *A Theory of Justice*:

‘Justice is the first virtue of social institutions, as truth is of systems of thought. A theory however elegant and economical must be rejected or revised if it is untrue; likewise, laws and institutions no matter how efficient and well-arranged must be reformed or abolished if they are unjust.’ [Read then states:] The form of the central proposition along with the analogy starkly reveal Rawls’ ‘*modelling* of his project on science’ that ‘is indeed ‘grand’ and bold’ and may be called ‘*scientism*’ [20, p. 344].

## Democratic and rational language: Rawlsian roots undergirding principlism

If the scientific image of language—presuming a crystalline purity to language such that a clear definition of autonomy or justice grants objective access to these realities—lingers backstage for the principlism drama, Rawlsian ‘public reason’ and ‘reasonableness’ takes stage front and center as lead actor. The “godfather of bioethics” [21], John Rawls’ *Theory of Justice* emerged in 1971 during the nascent years of bioethics, and while Rawls’ “reflective equilibrium” focuses mainly on his theory of social contract, bioethicists have adopted reflective equilibrium as arguably *the* methodology for bioethics.^3^ As the earlier debates swirled surrounding principlism, Beauchamp and Childress adopted reflective equilibrium in the 4th edition of *Principles of Biomedical Ethics* [22, pp. 1–26], officially placing the language debates over principlism within the framework of reflective equilibrium.

Far from removing all controversy, however, adopting reflective equilibrium more aptly embraced theoretical and practical challenges by flexibly merging a plurality of methods and beliefs. Arras writes: “Within the method of reflective equilibrium, there are no privileged beliefs. Every belief is fair game for pruning or grafting in the service of more confidently held beliefs of the same or other kinds” [3, p. 176]. Casuists, principlists, social theorists, and more can unite across the isles within the claim of reflective equilibrium that all opposing methodologies have a place within a democratic assembly of ‘competent’ moral judges. In this way, Rawls follows Kantian themes of reason, autonomy, and self-respect [23, p. 398], and yet beyond Kant, Rawls alters course from the Enlightenment tradition that seeks to ground moral and political principles explicitly within discursive rationality. Rawls’ constructive language speaks of democracy, and this maneuver has proved pragmatically well-suited to the ambitions of bioethics [24, p. 352], even while the core similarities or differences between Rawls and Kant remain open to question.^4^

In other words, reflective equilibrium offers a path for the language of principlism to move simultaneously in multiple directions. Particular moralities such as Jewish moral norms located in the Talmudic tradition, or even professional moralities maintained in physician codes, are thought to stem from what is common within *reasonably balanced* morality [23, p. 5–6]. The common morality, though ‘thin,’ is fine alongside ‘thick’ particular moralities deeply rooted in traditions, for both of these poles become core ingredients within the coherentist melting pot of reflective equilibrium. This equilibrist brew sees no problem with the specific beliefs of an individual or community as long as they integrate within the broader commitments of the universal, common morality. Similarly, when balancing organ donation according to ‘the expected number of years of survival’ with a waiting list that grants equal opportunity to all individuals, Beauchamp and Childress advocate for an ongoing adjustment of beliefs that accounts for moral norms behind *both* of these needs. Moreover, acknowledging that an ideal state of reflective equilibrium is not possible, Beauchamp and Childress pivot to the more limited goal of “trimming, repairing, and reshaping” beliefs in a realistic but not “utopian vision” process focused on “particular moralities” that are “continuously under development, not finished products” [23, pp. 441–42, 425–58].

The strategic advantages of this need hardly be drawn out. This vision of bioethics radically moves from the bottom up, drawing together terms and language from within various pieces and parts of a democratic society to construct a charter of maximally inclusive ethics. Can there be a more American vision of reason and morality? Even so, looking deeper, an aspirational assessment of reflective equilibrium would seem apt for a qualified comparison with Socratic dialogue. After all, Socrates embraces the questioning process, seeking knowledge and understanding via dialogue (*dia–logos*) amidst well-ordered first principles (*arx axia)*. Even while Rawls would reject Platonism as a “comprehensive doctrine,” might a highly optimistic Rawlisan pine for a comparative pathway to the Socrates dialogue? Both share in common a hope that through reasoned words (*logos*) a well-ordered politics may emerge. Aspirations aside, however, the comparison fails quickly if only given that the Socratic philosopher-king is a far cry from Rawl’s *citizen as citizen* who is bound by an immanent “public reason,” a reason that “belongs to a conception of a well ordered constitutional democratic society. The form and content of this reason…is part of the idea of democracy itself” [25, p. 765]. Even further from the Socratic dialogue moved by transcendent love as seen in the *Phaedrus*, Rawlsian public reason “neither criticizes nor attacks any comprehensive doctrine, religious or nonreligious, except insofar as that doctrine is incompatible with the essentials of public reason and a democratic polity” [25, pp. 765–766].

Put succinctly, the crux of this Rawlsian equilibrium substitutes “comprehensive doctrines of truth or right” for an “idea of the politically reasonable addressed to citizens as citizens” [25, p. 766]. In other words, public reason operates on the grounds of a “constitutional democratic regime” and “legitimate law,” and this is the politically liberal ideal. Applied to bioethics, the language of principlism takes meaning within the framework of free and equal citizens, making the boundaries of citizenship and public policy and law the determining factors. A Presidential Commission for the Study of Bioethical Issues has not been convened since 2017, and yet the Commission aptly represents the alignment of public reason and politics; charges against ideas of a previous Commission’s being too conservative or too progressive alike miss the nature of public reason. Bioethics *is* biopolitics, though an optimistic Rawlsian would surely wish to remove the Foucauldian critique associated with biopolitics, rendering the term with essentially positive connotations.

This brief summary of public reason and reflective equilibrium, however, has emphasized an aspirational vision of Rawlsian language. Amidst the claims that public reason is advantageous because it shortcuts altogether the quagmire of “metaphysical or philosophical or religious questions” [26, p. 9], one is left with a focus on *practical* matters. However, what resolutions actually arise within this practicality? What are the implications of the Kantian styled a priori conditions—e.g., original position and veil of ignorance—that lurk behind the promises of reflective equilibrium as right and correct reasoning? What *ordo* might guide these democratic discourses? Liberal members of society reason and engage *as citizens* with one another, yet one cannot miss that the humanity of a citizen *as such* is reduced to the value of the number one within some greater number [27, pp. 24–33]. Resorting to a single being as an individual citizen equals just one vote at the end of unresolved discourse. Rawlsian public reason assumes public voting, recalling de Tocqueville’s ‘tyranny of the majority,’ not to mention the collectivist rebuttals from Charles Taylor or Alasdair MacIntyre, and yet the momentum of reflective bioethical equilibrium seems unabated.

More specifically, public reason, at best, seems to offer a generalized, ‘ameliorative’ strategy that is dubiously equipped to resolve fundamental problems such as excessive health costs, definitions of death for organ transplantation, excess IVF embryos, etc. [28]. What is the amelioration within public reason that is grounded within the immanence of the public *qua* public? The Socratic critique of democracy certainly remains pertinent, and yet the more apt context for critiquing (or at least seeking to understand) the type of democratic impetus that informs Rawlsian public reason seems to stem from Rousseau’s “general will,” a will that “served as an abstract standard of judgement, an ideal of justice, a principle that provides a norm against which all considerations of individual or group interests must be measured. By definition, ‘the general will is always in favor of the common good’” [29, p. 27]. So, if, public reason ‘ameliorates’ bioethical challenges by revealing the common good, how is this formula not an equivocation between ‘general will’ and ‘common good’ that inaugurates a circular language system?

To be fair to Rawls, this circularity could be seen as an implicit feature more than a critique. Building on Chomsky’s theory of language, or universal grammar, Rawls sees a parallel between “moral cognition” and “universal grammar,” comparing his sense of justice to universal linguistic competence [30, pp. 46–53, 24]. This analogy places one firmly within moral psychology, for the core assumption behind justice, and by extension reflective equilibrium, is that humans are equipped with some innate moral framework. In the same way that language assumes a ‘universal grammar’ whereby people employ forms of speech all the time without reflecting on grammar, so, too, *some* moral intuition remains unconsciously in play. Phelan summarizes the argument, stating: “the observation that competent users of a natural language can construct an infinitely large set of novel utterances that are immediately acceptable to other speakers of the language. At the same time, such competent language users can assess whether an unbounded set of novel utterances produced by others are well—or ill—formed” [31, par. “The reasons for this supposition rest on analogies”].

In other words, Rawls borrows the pattern of Chomsky’s universal grammar to posit the possibility of moral reason amongst citizens because of an innate framework or ordo inherent within persons. The hinge, of course, is the ‘well or ill formed’ qualifier. Hence, when Beauchamp and Childress dismiss the Pirates Code of Ethics of the Brothers of the Coast of 1640 as a “moral outrage” albeit “internally coherent,” the primary justification for this judgement is the “need to cast the [reflective equilibrium] net more broadly” [22, p. 443]. This granted, what remains is to ponder the ‘what if’ when the majority of a population follows Bolshevik ethical tactics in the wake of Stalin, or when the majority supports the Aztec Empire governed by Nahuan metaphysics? How then does one find equilibrium amidst Stalin’s genocide or Aztec moral demands of human sacrifice? It is easy to dismiss moral intuitions and codes when the reflective net narrowly encircles Jehovah’s Witnesses and organized piracy, but do the Bolsheviks or Aztecs not meet the criteria of a wider net? If a universal grammar allows for manifold languages to cohere in some way (the very possibility of translation) can the framework of reflective equilibrium also enable moral translation in a liberal, democratic way alongside cultures that promote human sacrifice?

Some of these questions are beyond the scope of the argument here, and yet these puzzles help illustrate the expectations and presumptions of language and rationality. Rawlsian equilibrium asserts a ‘bottom up,’ reflective rationality amongst citizens that seems to offer bioethics “one method to rule them all” [3, p. 175], and yet the parallel with Chomsky’s grammar seems likely to bother those in bioethics who prefer attention only to procedural, democratic methods as opposed to the root questions of moral psychology. In other words, one must squarely face the question of whether Rawls’ own linguistic analogy enacts a foundational chink in the pragmatic armor of reflective equilibrium.

## Ascetic language: drawing Wittgenstein’s insights into ethics

Language, theorizing, and rationality are all too easily taken for granted. The picture I have described thus far seeks to highlight two approaches in bioethics that reveal approaches to language, theorizing, and rationality that move forward in the same groove. I see Kant as the champion of those who presume language and theories operate with crystalline, scientific clarity, and in a way, we might see him as the Enlightenment ‘ghost of bioethics’ past.’ This is a bioethics by obligation and duty. Rawls, then, is the champion for the liberal cosmopolitanism of bioethics’ ‘now,’ revealing that rationality and language are rightly suited for democratic reasoning. For Rawls, may the best argument win, where wins are determined by how wide the net of democratic acceptance is cast. These assumptions, however, bring me back to Davis’ argument that the principlism debates are more about rival “expectations for a ‘theory’” than they are about clearly articulated, substantive, definitions of autonomy, beneficence, and justice [10, pp. 103–104]. Again, the respective epistemological differences essentially guarantee agreement, yet the agreed upon conclusions are only accepted within the confines of those already sharing respective “antecedent epistemological commitments” [10, p. 85].

Unfortunately, critically pointing out the challenges of modes and ambiguities of language and rationality is rarely the popular strategy as Wittgenstein knew all too well. Hence, James Edward’s quip in 1975: “Wittgenstein is long dead, and philosophers have recovered their nerve” [32, p. 2]. In the name of progress, systematic philosophy seeks advances through theories or theses, and it becomes increasingly difficult to pause over Davis’ warning. When deeper, foundational issues remain unsettled, or exist amidst epistemological ambiguity, it is too soon to frame the walls and roof with systematic, constructive philosophies, and Wittgenstein saw this: “If I were to advance theses in philosophy, it would never be possible to debate them, because everyone would agree to them” [1, PI 128].

Wittgenstein’s linguistic analysis runs akin to phenomenology by distinguishing sense from nonsense in contrast to scientific philosophy—even physics—that seeks to determine true or false: “physics does not yield a description of the structure of phenomenological states of affairs. In phenomenology, it is always a matter of possibility, i.e., of sense, not of truth and falsity” [33, p. 63]. This phenomenological approach surprises those who only know Wittgenstein’s positivism in the *Tractatus*, but the arc of his philosophy, embracing both early and later Wittgenstein, seeks to clarify matters easily confused by language [16, pp. 46–55].^5^ For example, Wittgenstein addressed necessity and contingency by imagining that someone posits that a “circle” has the length of 3 cm and a width of 2 cm. The natural response, of course, asks what then we mean by a circle? For Wittgenstein, the “rules” in such a conversation are provided by syntax, or, simply the “grammar” of language that in this case links an “‘internal connection between something’s being a circle and its having only one radius” [33, p. 78]. Wittgenstein’s point is that the rule for a circle having only one radius makes sense due to the syntax or the “grammar” of language and not some presumed a priori necessity of ‘what is’ a circle. Further, one infers and articulates human agreement when distinguishing between colors, such as a ‘red’ or ‘blue’ ball, and all of this directs one’s attention away from presumptions and expectations of true–false necessity and determinacy instead towards the vital role of syntax and grammar play within our thinking. As Ray Monk has put it:

I used at one time to say that, in order to get clear how a sentence is used, it was a good idea to ask oneself the question: “How would one try to verify such an assertion?” But that’s just one way among others of getting clear about the use of a word or sentence. For example, another question which it is often very useful to ask oneself is: “How is this word learned?” “How would one set about teaching a child this word?” But some people have turned this suggestion about asking for the verification into a dogma—as if I’d been advancing a theory about meaning [34, pp. 287–288, 283–285].

Put another way, language for Wittgenstein does not reveal an essential *underlying* order within logic and the world, for that relationship is more synthetically integrated. He writes: “All propositions of our colloquial language are actually, just as they are, logically completely in order. That simple thing which we ought to give here is not a model of the truth but the complete truth itself. (Our problems are not abstract but perhaps the most concrete that there are.)” [35, 5.5563]. Contrary to Kantian-styled transcendentals or to Rawlsian-styled immanent expectations of meaning in language and reason, Wittgenstein seeks clarity by noting the *uses* of language. His aims are humbly apophatic, aiming to begin at least by mapping the limits of language. Hence, the whole point of “language games” is to observe *different* and *multiple* systems of communication, and even further to demonstrate that “not everything that we call language” appears in these systems [1, PI 3]. Navigating language systems—ranging from *orders* to *expressions* to *names* to *qualified terms* to any other *kinds of words*—calls one to an honest and humbler starting point to trace one’s ways with words. Some will ask for more, no doubt, expecting language to encapsulate a transcendental repository of meaning within a theory, or perhaps to inaugurate some meaning via a metaphysics of immanence. To such reactions Wittgenstein offers encouragement.

Don’t let it bother you that [some] languages…consist only of orders…[or] are incomplete, ask yourself whether our own language is complete—whether it was so before the symbolism of chemistry and the notation of the infinitesimal calculus were incorporated into it; for these are, so to speak, suburbs of our language. (And how many houses or streets can be regarded as an ancient city: a maze of little streets and squares, of old and new houses, of houses with extensions from various periods, and all this surrounded by a multitude of new suburbs with straight and regular streets and uniform houses) [1, PI 18].

In other words, Wittgenstein aims to map the complicated contours of an ancient city rather than to inaugurate democratic liberal equilibrium or to encapsulate meaning from the metaphysical horizon. From a Wittgensteinian viewpoint, principlism is a language game, meaning that the *kinds* of words used in bioethics take root primarily within the community of those participating in the game. Beauchamp and Childress’ principlism, in other words, stands to have traction within the *Lebensform* of a liberal, progressive, Western society because of that respective way of life, a way that synthetically relates to and functions alongside the principlist system of language. The contingent meaning within a liberal cosmopolitan *Lebensform* will see elective abortion and euthanasia, for example, as first order rights of autonomy. To expect principlism to transfer univocally upon global bioethics, however, misses the clarifying Wittgensteinian insight that *Lebensformen* deserve as much, if not more, attention than discursive reasoning. In Ethiopia, Orthodox Christians and traditional Muslims, for instance, will articulate an entirely different understanding of autonomy as the freedom to live within, respectively, the self-sacrificial Christian morality of the cross or the beliefs and practices embodied in the Qur’an and Sunnah. Appositely, truth-telling in the contemporary West is of paramount importance, whereas in Confucianism the familist moral concerns reflect a different set of obligations that justify at times blatant physician deception [36].

Moreover, many assume a pejorative critique via language games as though ‘language game’ is an exclusively negative critique. In place of negative connotations, however, the framework of language games brings clarity and an alternative for employing language to seek a liberal equilibrium or to presume meaning on the transcendental horizon. How so? Beyond the critical aspects of language games, for Wittgenstein, language is an opening to see ethics and life on the whole as a series of spiritual or ascetic exercises. By spiritual exercises, one does well to engage particular traditional religions, but considering Wittgenstein’s generalism, the more fitting comparison is to draw on Hellenistic or Roman schools of philosophy that aligned ethics *as* spiritual exercises. Hadot draws out that these ancient schools of philosophy embraced asceticism as formation or education (*paideia*) where learning both to live and to die were focal points of their “supraethical” practices [37]. For the Stoics, “learning to dialogue” and “learning to read” were not presumptive acts that led one to advanced theorizing, say, with principles of bioethics; rather, such practices aimed at an ancient self-formative philosophy “in its original aspect: not as a theoretical construct, but as a method for training people to live and to look at the world in a new way. It is an attempt to transform mankind” [37, p. 107].

To be sure, my emphasis has been on the *expectations* that emerge from language games, and much more could be said regarding the actual *practices* informing bioethics that come to light from a Wittgensteinian analysis. Alluding to the above *Lebensformen*, be they particular religious or philosophical traditions, can only be a gesture in passing to draw attention to a way of ethics that engages language ascetically, which is also to say poetically. Language for Wittgenstein is an opportunity for growth, emergence (*poeisis*), and formation (*ascesis*), and Wittgenstein’s later aphorisms—the *Philosophical Investigations* or the *Tractatus*— in addition to his deep appreciation for mysticism starkly reveal the thin expectations Wittgenstein had for moral guidance via transcendental or democratic rationality.

## Conclusion

At the birth of bioethics, figures such as Fritz Jahr (1895–1953) and Van Rensselaer Potter (1911–2001) spoke in terms that resonated with the language expectations Wittgenstein expounds. While I am under no pretension that a Wittgensteinian approach to language and ethics is easily accessible to contemporary bioethics, Jahr and Potter at least remind that bioethics does not lack *any* resonance with metaethics or the themes of cosmology so prominent within ancient classical life where the lines between religious and philosophical thinking were blurry [38]. Nor do I wish to imply that broad appeals to *Lebensformen* and non–theorizing philosophy are exhaustively curative for ongoing bioethics debates; rather, the aim is to encourage bioethics to take the modes and ambiguities of language sincerely, and even to view language as a pedagogical gateway. Accordingly, if one follows the terms of a debate, one will doubtless discover endless antecedent epistemological assumptions to be explicated. Even as Kant and Rawls represent ‘approaches’ within the debates on principlism that presume so much with language, Wittgenstein’s message to bioethics is to uncover these presumptions, and in this spirit his metaphilosophy is dedicated towards unveiling modern philosophy’s fascination with theorizing with ambiguous words that are often engrained more with confusion than clarity. Principlism enacts this trend in high resolution, and while a Wittgensteinian reading need not dismiss principlism *in toto*, at the least Wittgenstein better equips one to grasp the greater or lesser *use*, the quality, of these four principles.

## Footnotes

1 PI 126 summarizes a great deal about Wittgenstein’s view of philosophy for the sake of clarity: “Philosophy just puts everything before us, and neither explains nor deduces anything.—Since everything lies open to view, there is nothing to explain. For whatever may be hidden is of no interest to us” [1, PI 126].

2 To be clear, my aim is not to collapse distinctions between the Kantian a priori structure of cognition and his moral or practical reason. Kant’s transcendental method subsequently leads to moral judgements that are enabled via something a priori. Kantian ethics, then, postulates values via practical reason. In the end, Kerr’s description of Kantian “magic” or my description of values “out there” may give the sense of describing objective realities even while they more so describe the purported fruits of a priori cognitive structures.

3 The method of reflective equilibrium aims to move beyond particular systems or traditions of moral discernment, intuitions, or other judgements that alone may not justify particular moral positions. Rawls’ wide reflective equilibrium seeks to incorporate numerous reasoned differences to consider “all possible descriptions to which one might plausibly conform one’s judgements together with all relevant philosophical arguments for them” [30, p. 49].

4 While offering a full summary of the relationship between Kant and Rawls is beyond this essay, I am indebted to an anonymous reviewer for pointing out that the relationship between Kant and Rawls is *antecedent* to the question of how Rawls modifies Kant. In short, the democratic framework necessary for reflective equilibrium assumes a preceding a priori rationality grounded upon the ‘original position’ thought experiment. Further, within this strategic experimental starting point, the ‘veil of ignorance’ seeks to move beyond irrelevant matters—e.g., race, ethnicity, gender, age, wealth, comprehensive beliefs, etc.—to focus exclusively on free and equal citizens. These features and more form Kantian a priori conditions of fair and democratic rationality, the sum of which is no small task, for within Rawlsian constructivist language is the presumption to rationalize an immensity of human experience.

5 Consider the following additional insight from Wittgenstein: “Philosophy is a battle against the bewitchment of our intelligence by means of language” [1, PI 109].
